# Are We Really Addressing the Core of Children’s Environmental Health?

**DOI:** 10.1289/ehp.0901441

**Published:** 2009-10

**Authors:** Nsedu Obot Witherspoon

**Affiliations:** Children’s Environmental Health Network, Washington, DC, E-mail:nobot@cehn.org

More than 3 million children < 5 years of age die each year from environment-related conditions, making the environment one of the most critical contributors to the > 10 million child deaths annually throughout the world [World Health Organization (WHO) 2005]. Some factors of susceptibility, including race, ethnicity, and socioeconomic status, are generally not well understood in the context of risk assessment. For children’s environmental health the focus is almost exclusively on age-related differences and exposure hazards, without much discussion of the myriad additional factors including nutritional status, preexisting health status, multiple exposures, and gender differences that can affect the life course of a child.

We take pride in the fact that children’s environmental health has made great strides in research, national and international advocacy, and the acknowledgement of children’s vulnerabilities in the development of some policy and regulatory mechanisms. In the 1970s, we witnessed an increase of 5–6 IQ points in the average intelligence of children as a direct result of removing lead from paint and gasoline (Landrigan 2009). In 1996, the enactment of the Food Quality Protection Act created a historic and comprehensive overhaul of the pesticides and food safety laws while also requiring for the first time stricter safety standards for infants and children.

Thanks to the range of emerging pediatric research into the effects of air pollution on respiratory disease, the impacts of mercury, lead, and other contaminants on behavior and cognitive development, and prenatal and early-life exposures on child development, a strong case for protecting our children is being made. Fundamental to the growth of this field is the constant and fearless advocacy for primary prevention in children’s health by nonprofit and public interest organizations, both nationally and globally.

Important areas of concern for child health include air pollution, infectious and vectorborne diseases, food and water safety, impacts of climate change, heavy metals, and the built environment such as child care, schools, and homes. Emerging issues such as exposure to mercury in fish, ADHD, and connection between environmental hazards and learning disabilities have also gained attention.

Yet we must ask ourselves: Will we really witness the necessary reductions in environmental threats to children’s health by continuing on the same course? When looking at the context of children’s environmental health globally, we must also consider the importance of the underlying determinants of disease. The harsh inequities present in communities globally—poverty, lack of access to education, gender discrimination, lack of nutrition, and racism—hold vast implications for the health of children.

The wealthiest nations do not necessarily have the healthiest people (Shah 2009a). Rather, it is countries with the smallest economic gap between the rich and poor that have the healthiest populations. The United Nations Habitat’s State of the World’s Cities 2008/2009 report (UN-HABITAT 2008) found that with economic growth, high levels of inequity actually increase the likelihood of health disparities. Compared to all industrialized nations, the United States has the largest gap of inequality between rich and poor populations. The Latin American region has some of the highest income inequality between rich and poor populations (Gasparini et al. 2009).

Worldwide, environmental exposures contribute to the five major causes of death and illness among children < 5 years of age (WHO 2003): perinatal illness, respiratory disease, diarrheal diseases, vectorborne diseases, and physical injuries. Poverty and lack of nutrition are the common risk factors linking to all of these diseases.

Poorer child health is associated with the increasing number of children in poverty (Malat et al. 2005). Even with increased median household income, between 2006 and 2007, the child poverty rate in the United States reached the highest level since 1998, 18% (Mather 2008). Much of this increase was a result of worsening conditions among Latino and African-American families. The poorest tend to be more exposed to environmental hazards such as inadequate housing and proximity to polluting industries, resulting in higher levels of indoor and outdoor air pollutants and, consequently, respiratory disease. Annually, indoor air pollution from the use of solid fuels kills 1.5 million people; more than half are < 5 years of age (Shah 2009b)—a figure exceeding total deaths from malaria. Poverty presents challenges to overcoming risks through lack of resources and remoteness to necessary services.

Education is considered key to improving one’s life. Children who survive a variety of health threats before reaching school age find that nutrition and health affect their education, particularly their chances of enrollment (Jukes 2007). Some children get sick from the schools they attend, leading to increased absenteeism. Of the 2.2 billion children in the world, 1 billion live in poverty; 121 million are not enrolled in any form of education, and there is a loss of 443 million school days each year just from water-related illness (United Nations 2006). In Madagascar, for example, most schools do not have access to running water, contributing greatly to the lack of hygiene and sanitation facing children on this island. (Saholiariosa 2009). Due to the lack of sanitation, over half of children < 5 years of age in Madagascar die due to diarrhea. This trend is seen among the youngest children in nations with similar hygiene and sanitation challenges all over the globe.

Although gender discrimination has been officially banned for > 30 years in the United States, women around the world still are faced with this disparity and the resulting effects on their health and that of their children. In many Third World households, sons and daughters may be treated differently (Osafo-Kwaako 2001). Other sources of bias identified include low health and nutrition status of girls compared to boys and pro-male biases in wages (Horn and Beal 2004). Women who are employed in unregulated workplaces have the potential for increased exposure to hazards and lack of effective mechanisms for worker safety (WHO 2003), with particular dangers for fetuses.

There is growing recognition of the disparities children of color receive when it comes to their health care. Equity in health is an ethical and social justice issue. Adequately addressing the obvious inequities that children of racial and ethnic groups endure will improve the health care and health status of all children (Horn and Beal 2004). In the United States, a recent multicenter study found that of 5,147 fifth-graders 20% of African-American, 15% of Hispanic, and 15% of children identified as “other” reported perceived racial or ethnic discrimination (Tuzik, in press). Of those children, most were likely also to have symptoms of one or more of four mental health disorders: depression, ADHD, oppositional defiant disorder, and conduct disorder (Tuzik, in press). In Canada, groups such as recent migrants and Aboriginal populations face decreased income, increased unemployment rates, increased persistent poverty level, reduced access to housing, and therefore higher health risks (Public Health Agency of Canada 2004), with children especially affected.

We cannot successfully address children’s environmental health issues without addressing these underlying determinants. These issues will be increasingly important as we witness the inevitable implications of continued global economic crisis and climate change. Climate change will alter the global environment and present major challenges to the health and welfare of children. Children in communities that are already disadvantaged will be the most harmed. Atmospheric changes associated with greenhouse gases will lead to respiratory diseases and immunosuppression. Increased injury and death from extreme weather events and natural disasters, increased rates of allergies, malnutrition, exposure to certain toxicants, and exposure to vectorborne diseases is expected. Displacement of water and food security and forced migration due to drought may lead to political unrest, increased stress to families, and disruption in education [Children’s Environmental Health Network (CEHN) 2009]. Currently, most efforts organized to address children’s environmental health are focused on protection from environmental toxicants specifically. With emerging science, increased advocacy, and steps toward creating protective policies, we will continue to move in the right direction. However, as long as the inequities that underline children’s health overall remain, we will fall short of complete advance in children’s environmental protection.

The global community has begun to make these necessary connections. Organized by the WHO, The Global Initiative on Children’s Environmental Health Indicators was launched at the World Summit on Sustainable Development in 2002. The objectives of this Initiative are to develop and promote use of children’s environmental health indicators, improve assessment of children’s environmental health and monitor success or failure of interventions, and facilitate the ability of policy makers to improve environmental conditions for children. This effort also supports the WHO’s Commission on Social Determinants of Health, which was established to support countries and global health partners in addressing the social factors that lead to poor health and inequities. Overall, the Commission is working to improve daily living conditions, address inequitable distribution of power, money, and resources, and measure and assess impact of actions.

There must be a conscious effort to move beyond environmental remediation strategies toward environmental health promotion efforts that are sustainable and explicitly designed to reduce social, environmental, and health inequalities among our children (Schultz and Northridge 2004). It is essential for decision makers at international and national levels to continue working together with researchers, nongovernmental organizations, communities, and families to identify the linkages between key environmental hazards and the variety of social determinants plaguing children worldwide.

## Figures and Tables

**Figure:**
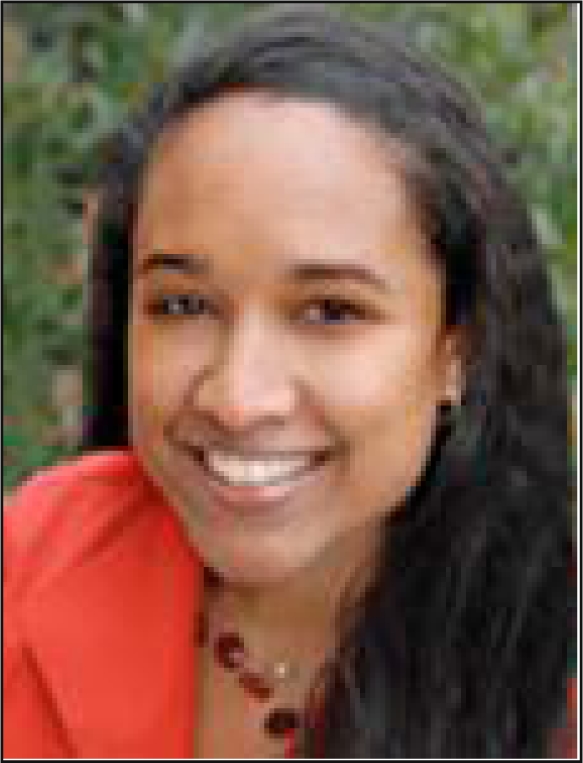
Nsedu Obot Witherspoon
